# Improved methods for detection of β-galactosidase (*lacZ*) activity in hard tissue

**DOI:** 10.1007/s00418-012-0936-1

**Published:** 2012-02-28

**Authors:** Akemi Shimada, Koichiro Komatsu, Kazuhisa Nakashima, Ernst Pöschl, Akira Nifuji

**Affiliations:** 1 Department of Pharmacology, School of Dental Medicine, Tsurumi University, 2-1-3 Tsurumi, Tsurumi-ku, Yokohama 230-8501 Japan; 2 School of Biological Sciences, University of East Anglia, Norwich, UK

**Keywords:** β-Galactosidase (*lacZ*), X-Gal, Hard tissue, Enzyme histochemistry, Fixation

## Abstract

The β-galactosidase gene (*lacZ*) of *Escherichia coli* is widely used as a reporter gene. The expression of *lacZ* can be detected by enzyme-based histochemical staining using chromogenic substrates such as 5-bromo-4-chloro-3-indolyl-β-d-galactoside (X-gal). Because the enzymatic activity of *lacZ* is vulnerable to high temperatures and acid treatment for demineralization, detection of *lacZ* on paraffinized sections is difficult, especially for hard tissues, which require demineralization before sectioning in paraffin. To circumvent this problem, whole-mount X-gal staining before sectioning is performed. However, detection of *lacZ* activity in the center of larger portions of hard whole adult tissues is challenging. In this study, focusing on fixation procedures, we determined the conditions conducive to improved detection of *lacZ* activity in deeper areas of whole tissues. We used an annexin a5 (*Anxa5*)-*lacZ* reporter mouse model in which the *Anxa5* expression in hard tissue is indicated by *lacZ* activity. We found that *lacZ* activity could be detected throughout the periodontal ligament of adult mice when fixed in 100% acetone, whereas it was not detected in the periodontal ligament around the root apex fixed in glutaraldehyde and paraformaldehyde. This staining could not be detected in wild-type mice. Acetone maintains the *lacZ* activity within 48 h of fixation at both 4°C and at room temperature. In conclusion, acetone is the optimal fixative to improve permeability for staining of *lacZ* activity in large volumes of adult hard tissues.

**Electronic supplementary material:**

The online version of this article (doi:10.1007/s00418-012-0936-1) contains supplementary material, which is available to authorized users.

## Introduction

Genetically engineered mice are used to elucidate molecular functions of genes in vivo. In such animals, in situ biochemical assays assist in the detection of temporal and spatial expression patterns for engineered genes and proteins (e.g. Sanes et al. 1986, Houzelstein et al. 1997; Diamond et al. 2000; Lézot et al. 2000). In addition, cells labeled by engineered genes are used to distinguish between donor and recipient cells in cell tracing studies (e.g. Sanchez-Ramos et al. 2000; Lekic et al. 2001). The β-galactosidase gene (*lacZ*) of *Escherichia coli* is often used as a reporter gene for these purposes (Cui et al. 1994).

The expression of *lacZ* can be detected by enzyme histochemical staining using chromogenic substrates such as 5-bromo-4-chloro-3-indolyl-β-d-galactoside (X-gal), 5-bromo-indolyl-β-*O*-galactopyranoside (Bluo-gal), and derivatives thereof (Aguzzi and Theuring 1994; Gioglio et al. 2002). β-Galactosidase hydrolyzes X-gal to form a highly sensitive blue precipitate. When histochemical staining is performed on the paraffinized section, the enzymatic activity is degraded by the high temperatures applied during the process of tissue preparation. The *lacZ* activity is lost under incubation at temperatures above 50°C, but not below 42°C (Avé et al. 1997; Cho et al. 2010). Moreover, in sections of hard tissues such as tooth and/or bone, detection of *lacZ* is often difficult because these tissues require complete decalcification (demineralization) to prepare appropriate sections for histological analysis. Because acid demineralization agents tend to weaken *lacZ* activity, demineralization by ethylene diamine tetra-acetic acid (EDTA) and detection using frozen sections is performed for teeth, although the resolution of frozen sections is lower than that of paraffinized sections (Cho et al. 2010). To prevent these problems, tissues and embryos are stained by whole-mount X-gal staining before sectioning. Conditions for detection of *lacZ* activity in different fixatives and stains have been previously investigated, and the standard protocols have been established (Murti and Schimenti 1991; Sanchez-Ramos et al. 2000; Pereira 2001; Gioglio et al. 2002; Ma et al. 2002; Takahashi et al. 2003; Bell et al. 2005; Bolon 2008). Despite these protocols, it is difficult to specifically detect the *lacZ* activity in the deeper regions of larger tissues, especially in hard tissues. An appropriate protocol for detection of *lacZ* activity in whole hard tissues has not yet been established.

In this study, we determined the conditions conducive to improved detection of *lacZ* activity in deeper areas of hard tissues. We used an annexin a5 (*Anxa5*)-*lacZ* reporter mouse model (Brachvogel et al. 2001; Brachvogel et al. 2005), in which the *Anxa5*-*lacZ* gene is expressed in the periodontal ligament tissue between the alveolar bone and the teeth (Shimada et al. in preparation).

## Materials and methods

All animal experiments were approved by the Institutional Animal Care Committee and the Recombination DNA Experiment and Biosafety Committee of the Tsurumi University School of Dental Medicine.

### Tissue preparation

A total of 44 *Anxa5*-*lacZ* reporter mice (Brachvogel et al. 2001) aged 3–11 months were used. As a negative control, 12 wild-type C57BL/6J mice (CLEA Japan, Inc., Tokyo, Japan) of similar ages were used. Mice were killed by cervical dislocation. The composition of fixatives and the fixation conditions are shown in Table 1. In the preliminary study, we identified *Anxa5* expression in periodontal tissues. To confirm *Anxa5*-*lacZ* expression in periodontal tissues, extracted mandibular first molars with the adherent periodontal ligament were fixed in 100% acetone and 0.2% glutaraldehyde. For whole-mount X-gal staining in larger hard tissue, the maxillae of adult mice were examined. The maxillae were skinned, dissected into approximately 20-mm tall, 15-mm wide, and 10-mm thick sections, and fixed in acetone, glutaraldehyde, and paraformaldehyde (PFA). To test the tissue permeability of fixatives, the maxillae were fixed in acetone, glutaraldehyde, and PFA with or without removal of palatine mucosa before fixation. The other tissues were fixed in glutaraldehyde and irradiated in a microwave (MW) oven (glutaraldehyde-MW, Murti and Schimenti 1991).

**Table 1:** Fixation conditions

Conditions	Composition of fixatives	Temperature	Time (number of specimen)
Acetone	100% acetone	4°C	8, 24, and 48 h (5, 3, and 2)
Acetone-RT	100% acetone	Room temperature (RT)	8, 24, and 48 h (4, 3, and 2)
Glutaraldehyde	0.2% glutaraldehyde, 5 mM EGTA, 10 mM MgCl_2_, 100 mM NaH_2_PO_4_ (pH 7.3) (Ma et al. 2002)	4°C	2, 8, 24, and 48 h (5, 5, 3, and 2)
Glutaraldehyde-MW	0.2% glutaraldehyde, 5 mM EGTA, 10 mM MgCl_2_, 100 mM NaH_2_PO_4_ (pH 7.3) (Murti and Schimenti 1991)	4°C	2 h immersion after microwave irradiation for 10 s at 500 W for 3 times (3)
PFA	3% paraformaldehyde, 1.25 mM EGTA, 2 mM MgCl_2,_ 100 mM PIPES (pH 7.4) (Bolon 2008)	4°C	3, 8 h (4 and 3)

### Staining of *lacZ* activity, demineralization, and preparation of paraffinized sections

Fixed tissues were washed with a reaction buffer consisting of 100 mM sodium phosphate buffer (pH 7.3), 2 mM magnesium chloride, 0.02% Nonidet P-40, and 0.01% sodium deoxycholate. The tissues were then stained for *lacZ* activity with a reaction buffer containing 1 mg/ml X-gal, 5 mM potassium ferricyanide, 5 mM potassium ferrocyanide, and 20 mM Tris-hydrochloric acid (pH 7.3). After staining, tissues were post-fixed in 4% PFA for 24 h at 4°C and demineralized with Morse’s solution (22.5% formic acid and 10% sodium citrate) for 36 h. Demineralized specimens were dehydrated in graded ethanol, cleared in Tissue-Tek^®^ Tissue-Clear™ (Sakura Finetek Japan, Tokyo, Japan), and embedded in Tissue-Tek^®^ Xpress^®^ Paraffin Wax II60 (Sakura Finetek Japan, Tokyo, Japan). Sagittal sections of maxillary first molars, with a thickness of 6 μm, were placed on slides pre-coated with aminopropyltriethoxysilane (Matsunami glass, Osaka, Japan).

### Assessment of *Anxa5*-*lacZ* detection

Teeth that were stained after extraction were photographed using a digital camera (DP71, Olympus, Tokyo, Japan) connected to a stereomicroscope (MZFLIII, Leica Microsystems Japan, Tokyo, Japan). Stained sections were photographed using a digital camera connected to a differential interference microscope (Eclipse 80i, Nikon, Tokyo, Japan). The area stained with X-gal in rectangular area measuring 65 μm wide and 380 μm tall including periodontal ligament in mesial side of mesial root and distal side of distal root was measured by ImageJ 1.39 (NIH, USA) (Fig. 2e). The stained area was calculated as percent area against total area and the difference of percent area among fixation conditions were statistically analyzed by Scheffe’s test. The fixation condition which shows the most extensive X-gal staining was assessed as optimal.

## Results

### Whole-mount *lacZ* staining of the periodontal tissue attached to an extracted tooth

In our preliminary experiment, we observed *Anxa5* expression in the periodontal tissue. Using *Anxa5*-*lacZ* knock-in mice, we extracted first molars of the mandible and examined *lacZ* expression as whole mounts. Extracted teeth were fixed in glutaraldehyde or acetone for 8 h at 4°C and whole teeth containing periodontal ligament tissues were stained with X-gal. Periodontal ligaments attached to tooth roots from *Anxa5*-*lacZ* mice showed strong blue staining (Supplemental Fig. 1c, d). This was not observed in the wild-type C57BL/6J mice (Supplemental Fig. 1a, b). The distribution of blue staining by X-gal was the same under both sets of fixation conditions. These results indicate that *lacZ* is uniformly stained under both conditions when tissues were directly exposed to fixative reagents.

### Optimal conditions for fixation for whole-mount *lacZ* staining of the maxillae

**Fig. 1:**
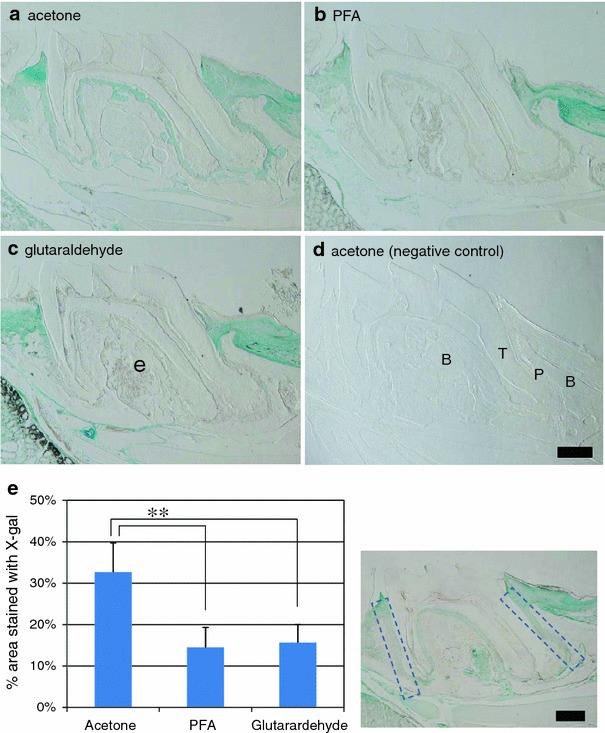
Optimal fixation conditions for detection of the *Anxa5*-*lacZ* gene in whole tissue. Maxillae from *Anxa5*-*lacZ* (**a**–**c**) and wild C57BL/6J (**d**) mice were fixed in acetone (**a** and **d**), PFA (**b**), and glutaraldehyde (**c**) for 8 h at 4°C. Fixed tissues were stained with X-gal for 5 days at 4°C, postfixed in PFA for 24 h at 4°C, demineralized with Morse’s solution for 36 h at 4°C, and embedded in paraffin. Six-micrometer-thick paraffin sections were prepared. Acetone fixed tissue (**a**) showed *Anxa5*-*lacZ* gene expression throughout the periodontal tissue and apical region of dental pulp. In the PFA (**b**) and glutaraldehyde (**c**) fixed tissues, the *Anxa5*-*lacZ* gene was not detected in the deeper area around the root apices. In the wild-type mice (**d**), the X-gal signal was not detected. *B* bone, *P* periodontal ligament, *T* tooth root. *Scale bar* 100 μm. (**e**) Percent area stained with X-gal (*left*) in rectangular areas (*right*) including mesial and distal sides of periodontal ligament after fixed with acetone, PFA, and glutaraldehyde. Mean ± SD **p < 0.01 (Scheffe’s test)

We next examined if *lacZ* expression in the periodontal ligament could be detected when we stained maxillary bone and teeth as whole maxillary tissues. Maxillae from *Anxa5*-*lacZ* mice were fixed under various fixative conditions (Table 1), stained with X-gal, postfixed in PFA, demineralized with Morse’s solution, and embedded in paraffin, according to the conventional procedure. In the PFA (Fig. 1b) and glutaraldehyde (Fig. 1c) fixed tissues, *lacZ* activity could be detected in palatine mucosa, gingival tissue, and the periodontal tissues only around the cervical area, and not around the root apices. In contrast, *lacZ* activity could be detected in all over the periodontal ligament tissues and apical part of dental pulp fixed with acetone (Fig. 1a). The mean percent area of X-gal staining in the specimen fixed with acetone was more than twice as large as those with other fixatives (Fig. 1e, *p* < 0.01). In the wild-type mice (Fig. 1d), blue staining of X-gal was not observed, indicating that endogenous β-galactosidase activity does not give rise to blue staining under our experimental conditions. These results suggest that acetone is the most effective fixative for detecting *lacZ* activity in deeper areas of maxillary tissue.

### Permeability of fixatives affects whole-mount staining of *lacZ*

**Fig. 2:**
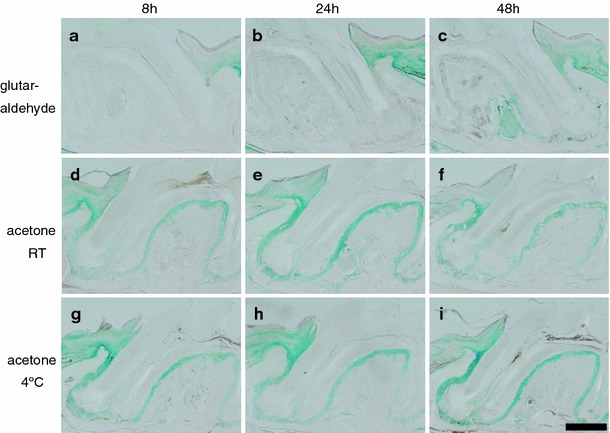
Effects of temperature and time of fixation. Maxillae from *Anxa5*-*lacZ* mice were fixed in glutaraldehyde (**a**–**c**) and acetone (**d**–**i**) at room temperature (RT) (**a**–**f**) and 4°C (**g**–**i**) for 8 h (**a**, **d**, and **g**), 24 h (**b**, **e**, and **h**), and 48 h (**c**, **f**, and **i**). Fixed tissues were stained with X-gal. Six-micrometer-thick paraffin sections were prepared by the procedure described in the legend of Fig. 1. In glutaraldehyde fixed tissues (**a**–**c**), *Anxa5*-*lacZ* signals were detected more extensively in a fixation time-dependent manner. In acetone fixed tissues (**d**–**i**), marked differences were not detected in *Anxa5*-*lacZ* signals under all conditions, even at the lower temperature (**g**–**i**). *Scale bar* 200 μm

When we stained whole maxillary tissue, the stained regions differed according to the fixative used. We considered that the differences in staining among the different fixatives were due to their different tissue permeabilities. To clarify this, we experimentally removed the physical barrier of the maxillae, i.e., mucosal epithelium, to increase the permeability of the fixatives. When maxillae were fixed in PFA (Supplemental Fig. 2b) and glutaraldehyde (Supplemental Fig. 2c) after removal of the palatine mucosa, the *lacZ* activity could be detected extensively in the deeper areas of the periodontal tissues relative to the tissues treated without removal of the mucosa (Fig. 1b, c, respectively). In contrast, the tissues fixed in acetone did not show a significant difference of area stained with X-gal before or after removal of the mucosa (Fig. 1a, Supplemental Fig. 2a). Irrespective of fixation conditions, intensity of X-gal staining in the periodontal ligament around cervical area increased after removal of palatine mucosa compared with opposite side of maxillae without removal of palatine mucosa. This increased *lacZ* activity could be due to the increased expression of annexin a5 protein, which was confirmed by immunohistochemical detection using annexin V antibody (Supplemental Fig. 3). When maxillae were fixed in glutaraldehyde and irradiated in the microwave oven without removal of the palatine mucosa (Supplemental Fig. 2d), *lacZ* activity was detected in the periodontal tissues around root apices, although the signal was relatively weak in deeper areas. When maxillae were fixed in glutaraldehyde for a longer time, the X-gal staining was expanded to deeper areas in the periodontal ligament (Fig. 2b, c). Taken together, these results indicate that the improved permeability induced by the fixatives allows us to detect *lacZ* activity in deeper areas of the periodontal ligament.

In general, the permeability of fixatives into tissue becomes lower at low temperatures, whereas enzymatic activity can be effectively retained at low temperatures. In contrast, excessive fixation conditions, such as the use of higher concentrations of fixatives and/or fixation for a longer time, may cause loss of enzymatic activity. Therefore, we sought to determine the optimal time and temperature conditions by varying these parameters for the process of fixation using acetone. When maxillae were fixed in acetone for 8, 24, and 48 h at 4°C (Fig. 2 g–i), *lacZ* activities detected in the periodontal tissues were found to be similar to those measured at room temperature (Fig. 2 d–f). The different fixation times ranging from 8 to 48 h did not produce a significant difference in *lacZ* activity with acetone (Fig. 2 d–i), but the maxillae fixed in acetone had significantly higher *lacZ* activity than maxillae fixed in glutaraldehyde under the same conditions of time and temperature (Fig. 2 a–c).

As a result, *Anxa5*-*lacZ* expressing cells could be clearly detected in the periodontal ligament around the root apex of tissues fixed in acetone for 8 h at 4°C (Supplemental Fig. 4a). Many *Anxa5*-*lacZ* expressing cells within the periodontal ligament tended to be located in perivascular sites (Supplemental Fig. 4b). Strong *Anxa5*-*lacZ* signals were also observed on the surface of alveolar bone (Supplemental Fig. 4b).

## Discussion

Various improved methods for histological observation of *lacZ*-expressing cells have been reported, including (a) staining with chromogenic substrates after sectioning under maintenance of *lacZ* enzymatic activity, (b) immunohistological detection of *lacZ* antigen after sectioning, and (c) sectioning after staining of *lacZ* activity with chromogenic substrates. Because demineralization using acid and paraffinization at high temperature causes loss of *lacZ* enzymatic activity, detection of *lacZ* activity specifically in hard tissue requires additional improvements to these protocols.

As the first method, Avé et al. (1997) succeeded in detecting *lacZ* activity in a paraffinized section of an embryo using low-melting point paraffin at 42°C. This method was less sensitive at 56°C. Cho et al. (2010) reported an improved method for rapid demineralization using 0.1 M EDTA at 42°C without loss of *lacZ* activity in frozen sections of hard tissues. The time required for complete demineralization of the skull of a 3-month-old mouse was 5–6 days in this protocol. The low-melting point paraffin, frozen sectioning, and demineralization at higher temperature have the disadvantage of requiring thin sectioning and tissue preservation for higher resolution.

As the second method, Pereira et al. (2006) reported an almost twofold increase in *lacZ*-transfected dermal cell staining using immunohistochemistry relative to histochemical staining with Bluo-Gal. This method includes activation of antigenicity by procedures such as proteinase pretreatment and heating of the section on the glass slide. In cases of analysis of hard tissue, heating causes specific tissue damage to tooth dentine and the surrounding tissue (Shimada et al. 2008). It should be noted that certain antibodies react not only with bacterial β-galactosidase but also with endogenous β-galactosidase (Kurz et al. 2000). Histochemical *lacZ* staining can distinguish between two enzymes by differences in optimal pH (Bolon 2008).

We chose the third method to detect *lacZ* activity using whole-tissue staining. For this purpose, appropriate fixatives have been examined previously. Gioglio et al. (2002) reported that muscle tissue fixed in a mixture of 2.5% glutaraldehyde and 1% paraformaldehyde showed the highest *lacZ* activity. Ma et al. (2002) reported that 0.2% glutaraldehyde produced the most consistent and reliable results. Bolon (2008) compared fixation conditions, including content, concentration, time and temperature for whole tissues, including bone dissected from Rosa26 mice, which have a *lacZ*-containing transgene, and reported that fixation in 3% paraformaldehyde for 3 h at 4°C was optimal. Hannouche et al. (2007) reported that fixation using a mixture of 2% formaldehyde and 0.2% glutaraldehyde for 4 days at 4°C gave the best results for X-gal staining of both cell cultures and bone implanted with mesenchymal stem cells from a vimentin-*lacZ* knock-in mouse.

In this study, improvement of permeability with PFA and glutaraldehyde by experimental removal of mucosa allowed us to detect the *lacZ* activity in deeper areas of the periodontal tissue (Supplemental Fig. 2). Interestingly, increased expression of Anxa5 around cervical area of periodontal ligament after invasive removal of palatine mucosa could be detected by both X-gal staining (Supplemental Fig. 2a–c) and immunohistochemistry (Supplemental Fig. 3), although the mechanisms of increased expression of annexin a5 after removal of tissue are unknown. However, detection of *lacZ* activity in apical region of periodontal ligament was insufficient by fixation with PFA and glutaraldehyde. Improvement of detection of *lacZ* activity in deeper area of the periodontal tissues could be also observed by microwave irradiation (Supplemental Fig. 2d). These results suggest that permeable fixatives are effective for staining large whole tissues and are suitable for the hard tissue that is difficult to micro-dissect before demineralization. Takahashi et al. (2003) showed that 100% acetone produces stronger X-gal staining than 0.2% glutaraldehyde for fixation of frozen sections of rat skeletal muscle. Based on this report, we compared acetone with other fixatives and found the most optimal fixatives for detection of *lacZ* activity in deeper areas of the tissue. The staining would not be caused by endogenous β-galactosidase activity, such as osteoclasts (Odgren et al. 2006), because such staining was not detected in wild-type C57BL/6J mice. Moreover, the immunohistological distribution of annexin a5 protein showed similar results (Shimada et al., in preparation). Acetone could easily permeabilize the tissues even at lower temperatures. This suggests that this fixative is effective for retaining the antigenicity for multistaining in the immunohistological study. In fact, we detected injected bromodeoxyuridine by immunohistochemistry after staining of *lacZ* activity (Shimada et al., in preparation). In other cases, it might be possible that acetone causes tissue shrinkage and hardening or/and loss of antigenicity due to its lipid- and glycolipd-solubility. It has been reported that over-fixation causes a decrease in *lacZ* activity (Gioglio et al. 2002; Ma et al. 2002). However, acetone fixation within 48 h did not decrease the *lacZ* activity (Fig. 2), suggesting it is a milder fixative. Acetone must be exchanged frequently to complete the fixation process. We suggest that incomplete fixation caused diffuse staining with X-gal (data not shown). It also needs to be careful for staining specificity in the keratinized part of the mucosa after fixation with acetone, although the distribution of annexin a5 could be detected in the keratinized part of palatine mucosa by immunohistochemical detection after fixation with PFA (Supplemental Fig. 3a).

In conclusion, permeable acetone is the optimal fixative for staining of *lacZ* activity in deep areas of whole hard tissues.

## Electronic supplementary material

Below is the link to the electronic supplementary material.

Supplementary material 1 (PPT 2156 kb)

Supplementary material 2 (DOC 43 kb)

## References

[CR1] Aguzzi A, Theuring F (1994). Improved in situ β-galactosidase staining for histological analysis of transgenic mice. Histochemistry.

[CR2] Avé P, Colucci-Guyon E, Babinet C, Huerre MR (1997). An improved method to detect β-galactosidase activity in transgenic mice: a post-staining procedure on paraffin embedded tissue sections. Transgenic Res.

[CR3] Bell P, Limberis M, Gao G, Wu D, Bove MS, Sanmiguel JC, Wilson JM (2005). An optimized protocol for detection of *E. coli* β-galactosidase in lung tissue following gene transfer. Histochem Cell Biol.

[CR4] Bolon B (2008). Whole mount enzyme histochemistry as a rapid screen at necropsy for expression of β-galactosidase (*LacZ*)-bearing transgenes: considerations for separating specific LacZ activity from nonspecific (endogenous) galactosidase activity. Toxicol Pathol.

[CR5] Brachvogel B, Welzel H, Moch H, von der Mark K, Hofmann C, Pöschl E (2001). Sequential expression of annexin A5 in the vasculature and skeletal elements during mouse development. Mech Dev.

[CR6] Brachvogel B, Moch H, Pausch F, Schlötzer-Schrehardt U, Hofmann C, Hallmann R, von der Mark K, Winkler T, Pöschl E (2005). Perivascular cells expressing annexin A5 define a novel mesenchymal stem cell-like population with the capacity to differentiate into multiple mesenchymal lineages. Development.

[CR7] Cho A, Suzuki S, Hatakeyama J, Haruyama N, Kulkarni AB (2010). A method for rapid demineralization of teeth and bones. Open Dent J.

[CR8] Cui C, Wani MA, Wight D, Kopchick J, Stambrook PJ (1994). Reporter genes in transgenic mice. Transgenic Res.

[CR9] Diamond I, Owolabi T, Marco M, Lam C, Glick A (2000). Conditional gene expression in the epidermis of transgenic mice using the tetracycline-regulated transactivators tTA and rTA linked to the keratin 5 promoter. J Invest Dermatol.

[CR10] Gioglio L, de Cusella AM, Boratto R, Poggi P (2002). An improved method for β-galactosidase activity detection on muscle tissue. A light and electron microscopic study. Ann Anat.

[CR11] Hannouche D, Raould A, Nizard RS, Sedel L, Petite H (2007). Embedding of bone samples in methylmethacrylate: a suitable method for tracking LacZ mesenchymal stem cells in skeletal tissues. J Histochem Cytochem.

[CR12] Houzelstein D, Cohen A, Buckingham ME, Robert B (1997). Insertional mutation of the mouse *Msx1* homeobox gene by an *nlacZ* reporter gene. Mech Dev.

[CR13] Kurz DJ, Decary S, Hong Y, Erusalimsky JD (2000). Senescence-associated β-galactosidase reflects an increase in lysosomal mass during replicative ageing of human endothelial cells. J Cell Sci.

[CR14] Lekic PC, Rajshankar D, Chen H, Tenenbaum H, McCulloch CAG (2001). Transplantation of labeled periodontal ligament cells promotes regeneration of alveolar bone. Anat Rec.

[CR15] Lézot F, Thomas B, Hotton D, Forest N, Orestes-Cardoso S, Robert B, Sharpe P, Berdal A (2000). Biomineralization, life-time of odontogenic cells and differential expression of the two homeobox genes *MSX*-*1* and *DLX*-*2* in transgenic mice. J Bone Miner Res.

[CR16] Ma W, Rogers K, Zbar B, Schmidt L (2002). Effects of different fixatives on β-galactosidase activity. J Histochem Cytochem.

[CR17] Murti JR, Schimenti JC (1991). Microwave-accelerated fixation and *lacZ* activity staining of testicular cells in transgenic mice. Anal Biochem.

[CR18] Odgren PR, MacKay CA, Mason-Savas A, Yang M, Mailhot G, Birnbaum MJ (2006). False-positive β-galactosidase staining in osteoclasts by endogenous enzyme: studies in neonatal and month-old wild-type mice. Connect Tissue Res.

[CR19] Pereira FA. (2001) Whole-mount histochemical detection of beta-galactosidase activity. In: Ausubel FM et al. (ed) Current protocols in molecular biology, Wiley-Interscience, New York, Chapter 14:Unit 14.14 10.1002/0471142727.mb1414s50 18265109

[CR20] Pereira C, Maamar-Tayeb M, Burke A, Perez-Polo R, Herndon DN, Jeschke MG (2006). Immunohistochemical staining of transgenic beta-galactosidase in burned skin is a better indicator of transfection efficiency than histochemical techniques. J Immunol Methods.

[CR21] Sanchez-Ramos J, Song S, Dailey M, Cardozo-Pelaez F, Hazzi C, Stedeford T, Willing A, Freeman TB, Saporta S, Zigova T, Sanberg PR, Snyder EY (2000). The X-gal caution in neural transplantation studies. Cell Transplant.

[CR22] Sanes JR, Rubenstein JL, Nicolas JF (1986). Use of a recombinant retrovirus to study post-implantation cell lineage in mouse embryos. EMBO J.

[CR23] Shimada A, Shibata T, Komatsu K, Nifuji A (2008). Improved methods for immunohistochemical detection of BrdU in hard tissue. J Immunol Methods.

[CR24] Takahashi M, Hakamata Y, Takeuchi K, Kobayashi E (2003). Effects of different fixatives on β-galactosidase activity. J Histochem Cytochem.

