# Supplementary material for: Improved methods for detection of β-galactosidase (lacZ) activity in hard tissue
Source: Histochem Cell Biol. 2012 Feb 28;137(6):841–7. doi: 10.1007/s00418-012-0936-1 (PMC3353101; doi:10.1007/s00418-012-0936-1)
Supplement: Supplementary file 1 — Supplementary material 1 (PPT 2156 kb) [file 418_2012_936_MOESM1_ESM.ppt]

## Slide 1
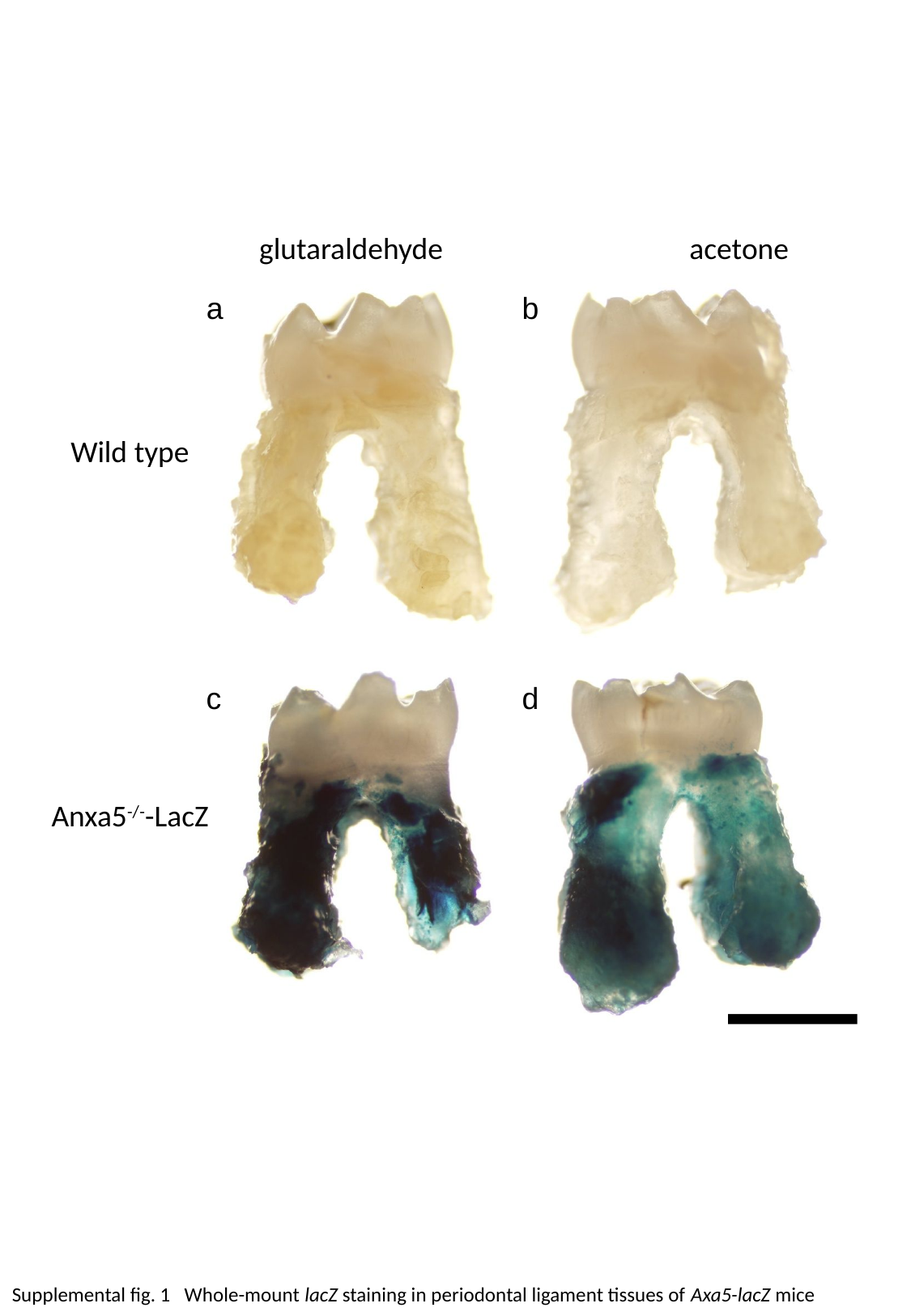

glutaraldehyde 　 acetone
a
b
c
d
Wild type
Anxa5-/--LacZ
# Supplemental fig. 1 Whole-mount lacZ staining in periodontal ligament tissues of Axa5-lacZ mice

## Slide 2
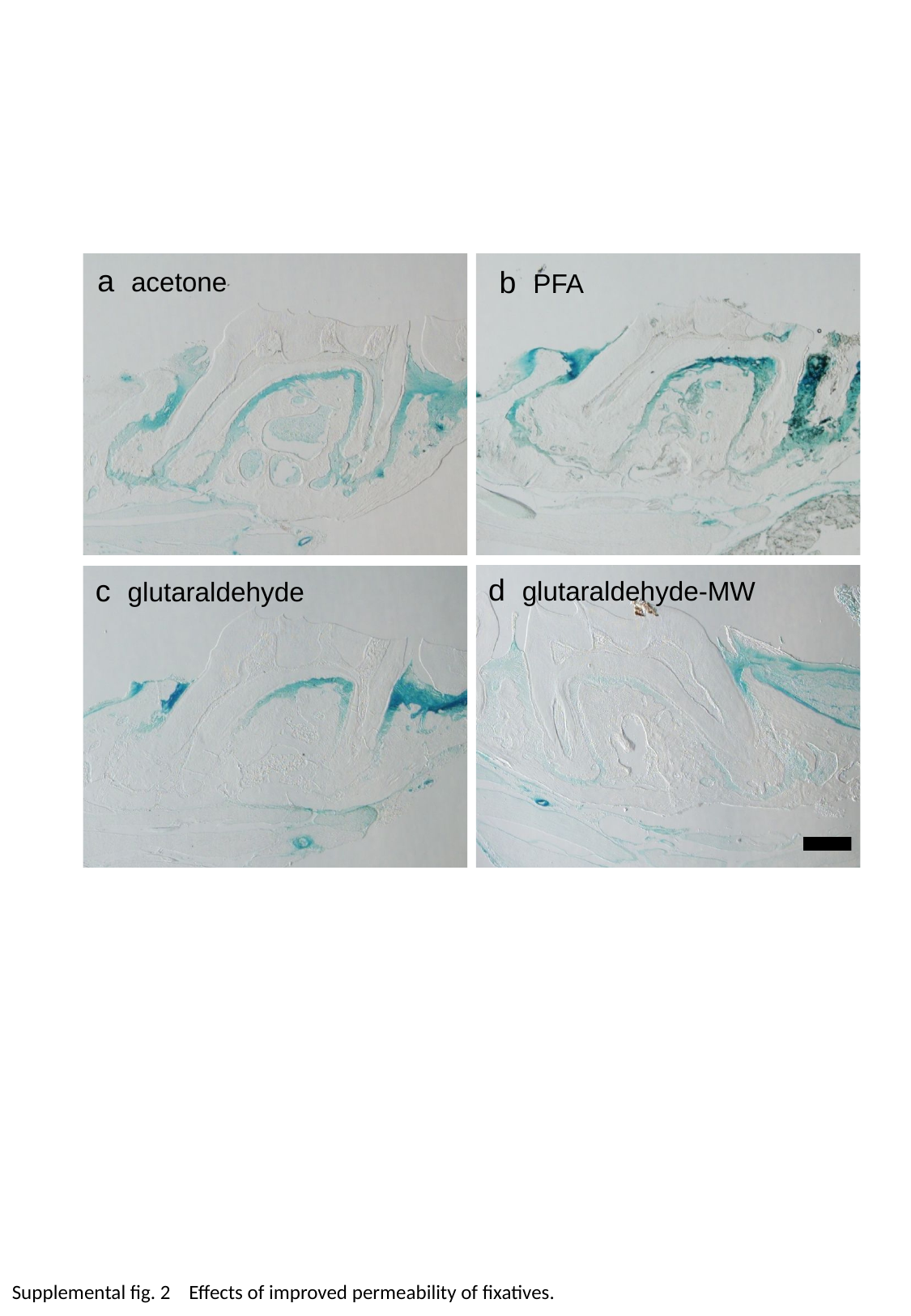

a acetone
b PFA
d glutaraldehyde-MW
c glutaraldehyde
# Supplemental fig. 2 Effects of improved permeability of fixatives.

## Slide 3
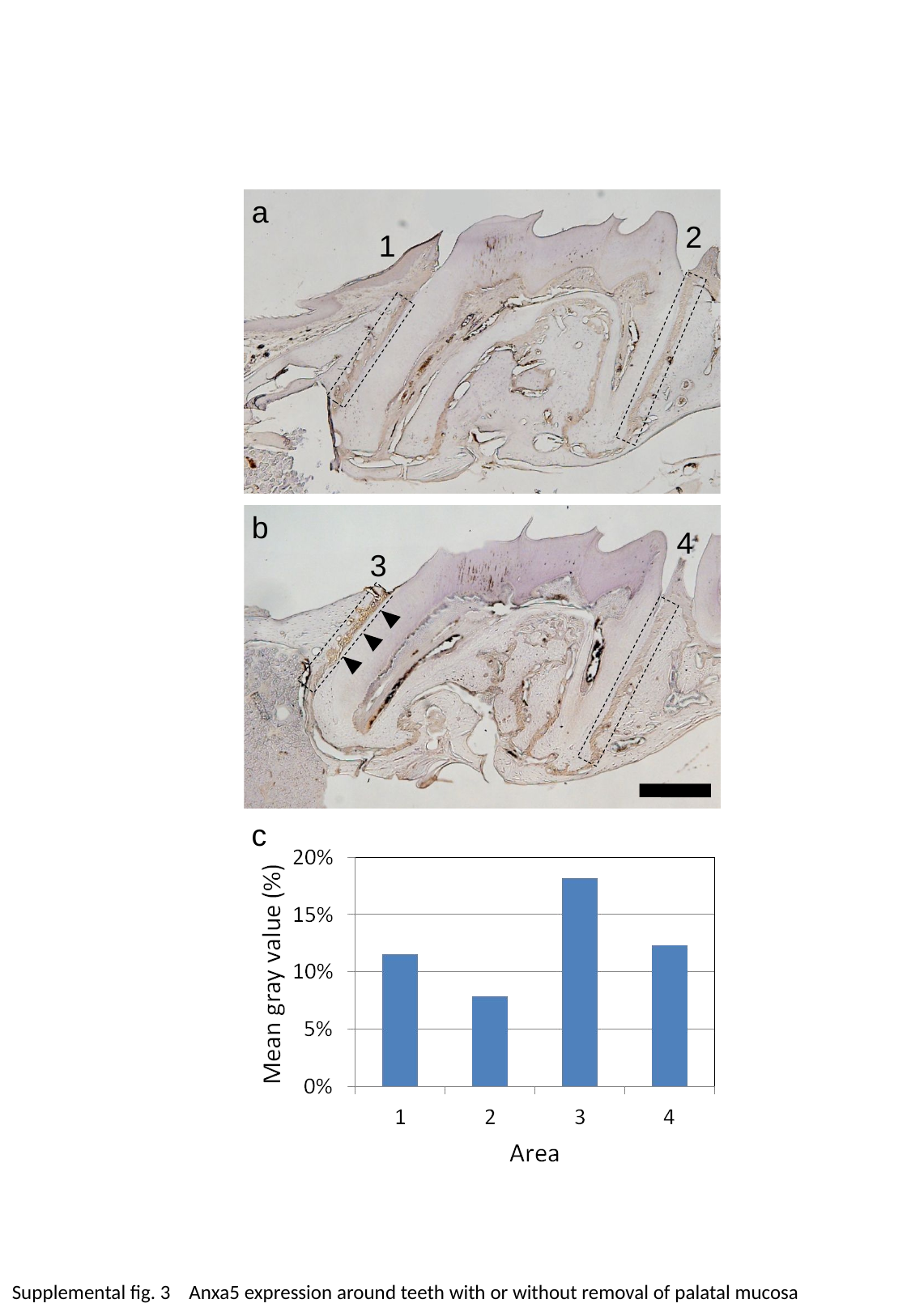

a
2
1
b
4
3
c
Supplemental fig. 3 Anxa5 expression around teeth with or without removal of palatal mucosa

## Slide 4
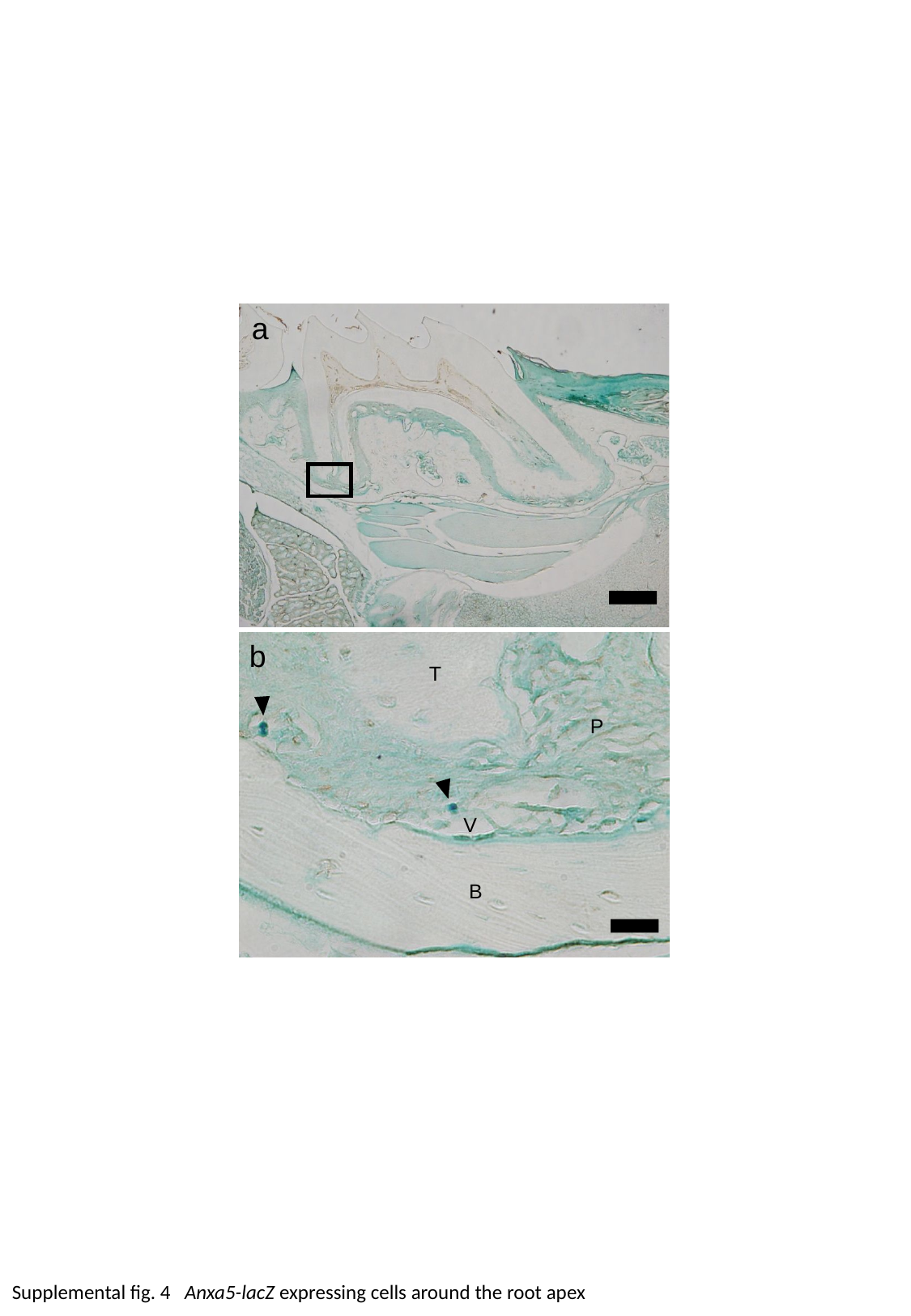

a
b
T
P
V
B
Supplemental fig. 4 Anxa5-lacZ expressing cells around the root apex
