# Supplementary material for: Improved methods for detection of β-galactosidase (lacZ) activity in hard tissue
Source: Histochem Cell Biol. 2012 Feb 28;137(6):841–7. doi: 10.1007/s00418-012-0936-1 (PMC3353101; doi:10.1007/s00418-012-0936-1)
Supplement: Supplementary file 2 — Supplementary material 2 (DOC 43 kb) [file 418_2012_936_MOESM2_ESM.doc]

Histochemistry and Cell Biology

Short communications

Improved methods for detection of β-galactosidase (*lacZ*) activity in hard tissue

Akemi Shimada^1*^, Koichiro Komatsu^1^, Kazuhisa Nakashima^1^, Ernst Pöschl^2^, Akira Nifuji^1*^

1. Department of Pharmacology, Tsurumi University School of Dental Medicine, Yokohama, Japan

2. School of Biological Sciences, University of East Anglia, Norwich, United Kingdom

Corresponding authors:

2-1-3 Tsurumi, Tsurumi-ku, Yokohama 230-8501, Japan,

Tel, +81-45-581-1001; Fax, +81-45-573-9599

E-mail; shimada-a@tsurumi-u.ac.jp (Akemi Shimada)

E-mail, nifuji-a@tsurumi-u.ac.jp (Akira Nifuji)

Supplemental information

**Materials and methods**

1. Immunohistochemistry

After cervical dislocation, the dissected maxilla of ICR mouse was divided into a right and a left half. To test the effect of invasion to expression of annexin a5, the maxillae with or without removal of palatine mucosa were fixed in 4% paraformaldehyde including phosphate buffered saline for 24 hours. Then, the tissues were demineralized with 20% ethylenediaminetetraacetic acid for 3 weeks, and embedded in paraffin in ordinary procedures. Sagittal sections (5 μm thickness) of the first molar were made. The expression of annexin a5 protein were examined with rabbit anti-human annexin V antibody (BioVision, Inc., CA, USA) and Histofine® Simple Stain Mouse MAX-PO (R) (Nichirei, Tokyo, Japan). Peroxidase reaction was developed by 3-3’-diaminobenzidine (DAB). The intensity of DAB staining was quantitatively compared mean gray values in rectangular areas including periodontal ligament of mesial side of mesial root and of distal side of distal root were measured by by ImageJ 1.39.

**Figure caption**

Supplemental fig. 1: Whole-mount *lacZ* staining in periodontal ligament tissues of Axa5-*lacZ* mice.

Mandibular first molars were extracted from wild C57BL/6J (a, b) and *Anxa5*-*lacZ* (c, d) mice. The extracted teeth were fixed in glutaraldehyde (a, c) and acetone (b, d) for 8 h at 4°C, and stained with X-gal as described in materials and methods. Periodontal ligament tissues adhered to the tooth roots from *Anxa5*-*lacZ* mice (c, d) showed *Anxa5*-*lacZ* gene expression under both fixation conditions. Expression was not observed in the wild-type mice (a, b). Scale bar = 1 mm.

Supplemental fig. 2: Effects of improved permeability of fixatives.

To improve the permeability of fixatives, maxillae were fixed in acetone (a), PFA (b) and glutaraldehyde (c) after removal of the palatine mucosa. Other maxillae were fixed in glutaraldehyde-MW (d) without removal of the palatine mucosa. Fixed tissues were stained with X-gal. Six-micrometer-thick paraffin sections were prepared by the same procedure described in the legend of Fig. 2. In acetone fixed tissue (a), the *Anxa5*-*lacZ* signals detected were similar to those detected without removal of mucosa (Fig. 2a). In PFA (b), glutaraldehyde (c), and glutaraldehyde-MW (d) fixed tissues, *Anxa5*-*lacZ* signals were detected more extensively in deeper areas of the periodontal ligament relative to tissues fixed without removal of mucosa and MW irradiation. Scale bar = 100 μm.

Supplemental fig. 3. Immunohistochemical detection of increased expression of annexin a5 after tissue removal.

(a, b) The expression of annexin a5 around maxillary first molars were compared between with (b) or without (a) removal of palatine mucosa. Brown staining of 3-3’-diaminobenzidine (DAB) could be detected in all periodontal ligament tissues. After removal of palatine mucosa (b), increased expression of annexin a5 around cervical area of periodontal ligament was observed (arrowheads). Four rectangles and those number indicate the areas measured intensity of DAB staining. Scale = 100 μm. (c) Mean gray values in 4 rectangls including periodontal ligament. Mean gray values were indexed from 0 at a gray level of background to 100 at a level of black.

Supplemental fig. 4: *Anxa5*-*lacZ* expressing cells around the root apex

Maxillae from *Anxa5*-*lacZ* mice were fixed in acetone at 4°C for 8 hours. Fixed tissues were stained with X-gal. Six-micrometer-thick paraffin sections were prepared by procedure described in the legend of Fig. 2. *Anxa5*-*lacZ* signals were detected around the root apices (b) indicated in a square area in the lower magnified photograph (a). Triangles indicate *Anxa5*-*lacZ* expressing perivascular cells within the periodontal ligament. B, bone; P, periodontal ligament; T, tooth root; V, blood vessel. Scale bars = 100 μm (a) and 5 μm (b).
